# ^68^Ga-bisphosphonates for the imaging of extraosseous calcification by positron emission tomography

**DOI:** 10.1038/s41598-023-41149-7

**Published:** 2023-09-05

**Authors:** George P. Keeling, Friedrich Baark, Orestis L. Katsamenis, Jing Xue, Philip J. Blower, Sergio Bertazzo, Rafael T. M. de Rosales

**Affiliations:** 1 Department of Imaging Chemistry & Biology, School of Biomedical Engineering & Imaging Sciences, King’s College London, St Thomas’ Hospital, London, SE1 7EH UK; 2 Faculty of Engineering and Physical Sciences, Highfield Campus, µ-VIS X-Ray Imaging Centre, University of Southampton, Southampton, SO17 1BJ UK; 3 Department of Medical Physics & Biomedical Engineering, University College London, Malet Place Engineering Building, London, WC1E 6BT UK

**Keywords:** Calcification, Chronic kidney disease, Molecular imaging, Positron-emission tomography, X-ray tomography

## Abstract

Radiolabelled bisphosphonates (BPs) and [^18^F]NaF (^18^F-fluoride) are the two types of radiotracers available to image calcium mineral (e.g. bone), yet only [^18^F]NaF has been widely explored for the non-invasive molecular imaging of extraosseous calcification (EC) using positron emission tomography (PET) imaging. These two radiotracers bind calcium mineral deposits via different mechanisms, with BPs chelating to calcium ions and thus being non-selective, and [^18^F]NaF being selective for hydroxyapatite (HAp) which is the main component of bone mineral. Considering that the composition of EC has been reported to include a diverse range of non-HAp calcium minerals, we hypothesised that BPs may be more sensitive for imaging EC due to their ability to bind to both HAp and non-HAp deposits. We report a comparison between the ^68^Ga-labelled BP tracer [^68^Ga]Ga-THP-Pam and [^18^F]NaF for PET imaging in a rat model of EC that develops macro- and microcalcifications in several organs. Macrocalcifications were identified using preclinical computed tomography (CT) and microcalcifications were identified using µCT-based 3D X-ray histology (XRH) on isolated organs ex vivo. The morphological and mineral analysis of individual calcified deposits was performed using scanning electron microscopy (SEM) and energy-dispersive X-ray spectroscopy (EDX). PET imaging and ex vivo analysis results demonstrated that while both radiotracers behave similarly for bone imaging, the BP-based radiotracer [^68^Ga]Ga-THP-Pam was able to detect EC more sensitively in several organs in which the mineral composition departs from that of HAp. Our results strongly suggest that BP-based PET radiotracers such as [^68^Ga]Ga-THP-Pam may have a particular advantage for the sensitive imaging and early detection of EC by being able to detect a wider array of relevant calcium minerals in vivo than [^18^F]NaF, and should be evaluated clinically for this purpose.

## Introduction

Bisphosphonates (BPs) are compounds with high affinity for solid calcium minerals such as hydroxyapatite (HAp)^1^, the primary inorganic component of bone tissue^2^. BPs accumulate particularly in areas of high mineral turnover, such as bone metastases^1,3^, and have been the mainstay of medical imaging of bone diseases since the 1970s, most commonly in the form of [^99m^Tc]Tc-MDP using gamma-scintigraphy/SPECT imaging^3–5^.

**Figure 1:**
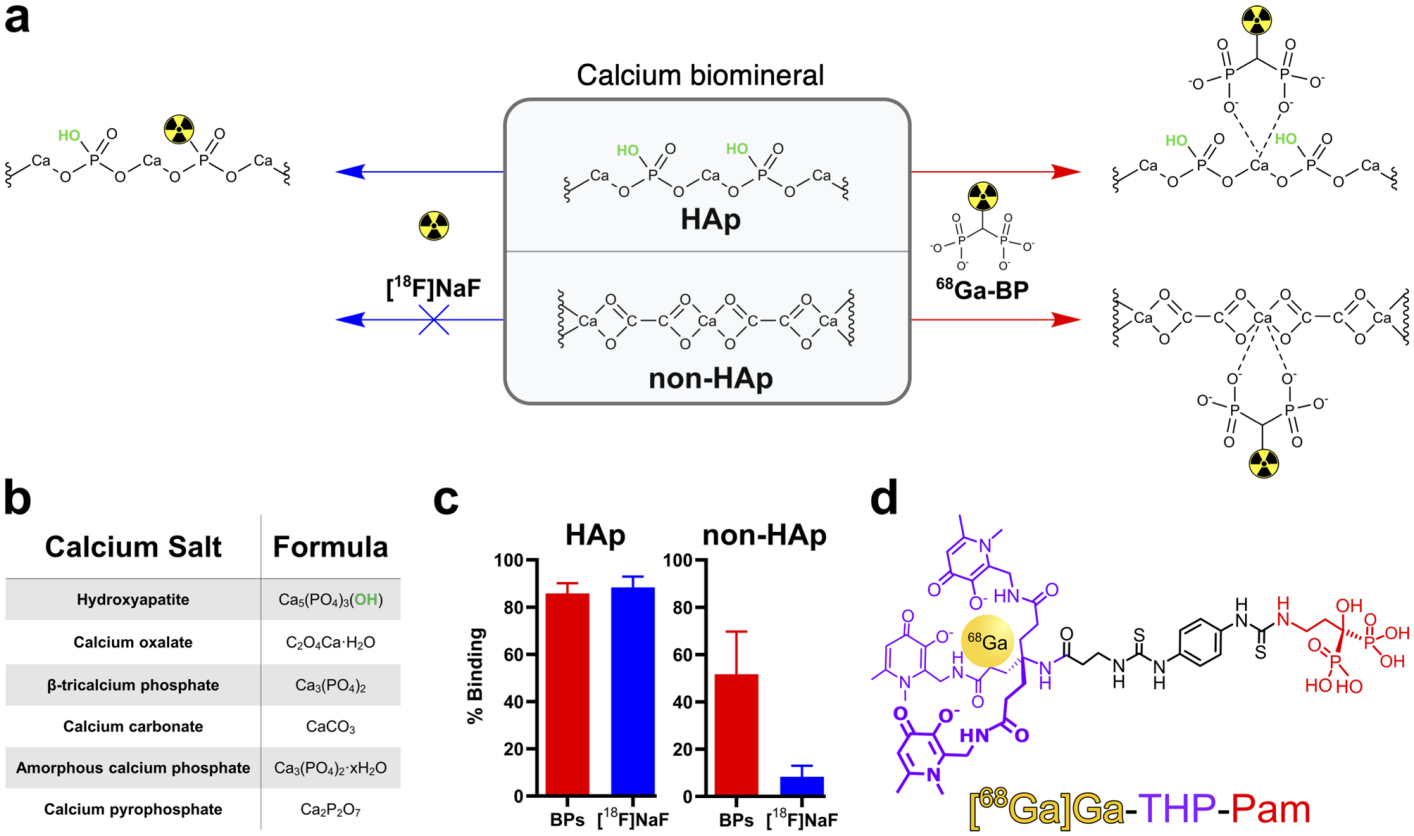
(**a**) Schematic showing the different binding modes of [^18^F]NaF and a generic BP to calcium salts, including the lack of reaction between [^18^F]NaF and salts without hydroxyl anions. (**b**) Formulae of selected biologically relevant calcium salts. (**c**) Summary of in vitro binding data of selected ^68^Ga-BPs presented in our previous work^17^ in comparison to [^18^F]NaF in HAp and the non-HAp salts listed in (**b**). (**d**) Structure of [^68^Ga]Ga-THP-Pam with the radiometal, gallium-68 shown in yellow; the chelator, THP, shown in purple; and the calcium-binding group, pamidronate, shown in red.

Technological advances in positron emission tomography (PET) have ushered in renewed interest in the development of BP-based imaging agents due to its high sensitivity and spatial resolution. In recent years, a flurry of new BP tracers using the generator-produced positron emitter ^68^Ga (*t*_*1/2*_ = 68 min), have been reported, mainly focusing on applications for the detection of bone metastases^6–17^. However, the current most-used tracer for the PET imaging of bone metastases in the clinic is [^18^F]NaF, which functions due to the fluoride ion’s ability to displace the hydroxyl group in the structure of HAp (Fig. 1a)^18^.

Previously, we have demonstrated that the binding mode of [^18^F]NaF leads to binding specificity towards HAp over other calcium minerals (Fig. 1a–c)^17^. However, BPs—which bind through the interaction of the phosphonate groups with calcium ions—have a much broader range of calcium mineral affinity^3,17,19^. The prevalence of HAp in bones makes [^18^F]NaF an excellent tracer for the imaging of bones. On the other hand, reports on the mineral composition of extraosseous calcification (EC), which is defined as “deposition of calcium in tissues outside of bone”^20^ and includes conditions such as vascular calcification, are diverse and contradictory^21–32^. These calcifications are often euphemistically called HAp as a blanket term for all forms of apatite or solid calcium-containing mineral. However, the general consensus in the literature is that early calcification begins as microcalcifications of amorphous calcium phosphate and whitlockite and, as the calcification progresses, HAp crystals develop and merge into larger sheets or plaques of macrocalcification^33^. However, this is dependent on a number of factors including the location of the calcification and its underlying cause^34^. This consensus is oversimplified and a range of calcium salts may be observed including HAp, apatite, (amorphous) calcium phosphate, whitlockite, calcium oxalate and calcium carbonate^34^. Various studies have demonstrated the presence of calcification in a wide variety of diseases, including atherosclerosis^25,29,30^, age-related macular degeneration^35^, Alzheimer’s disease^36^, muscular dystrophy^23^, various cancers^21,37,38^, kidney stones^26,27,31^ and chronic kidney disease (CKD)^39^.

Nonetheless, [^18^F]NaF is the only clinically used PET tracer for the imaging of extraosseous calcification^40^, although a recent study has demonstrated the feasibility of the PET BP [^68^Ga]Ga-NODAGA^ZOL^ for the imaging of EC in atherosclerotic plaques^41^. Given the potentially diverse forms of calcium mineral present in such calcification, we hypothesised that a HAp-selective tracer such as [^18^F]NaF is not the most appropriate imaging agent for a condition in which HAp may not be the most common form of calcium mineral. To test this hypothesis, we compared the performance of [^18^F]NaF with the BP-based conjugate of the ^68^Ga chelator *tris*(hydroxypyridinone) (THP)—[^68^Ga]Ga-THP-Pam (Fig. 1d)^17^, using a rat model that develops macro- and microcalcification across several major organs, and highlighted the differences in imaging results that stem from the mineral composition of the calcification.

## Results

### Preclinical PET/CT imaging in disease (extraosseous calcification diet—EC) and healthy animals

**Figure 2:**
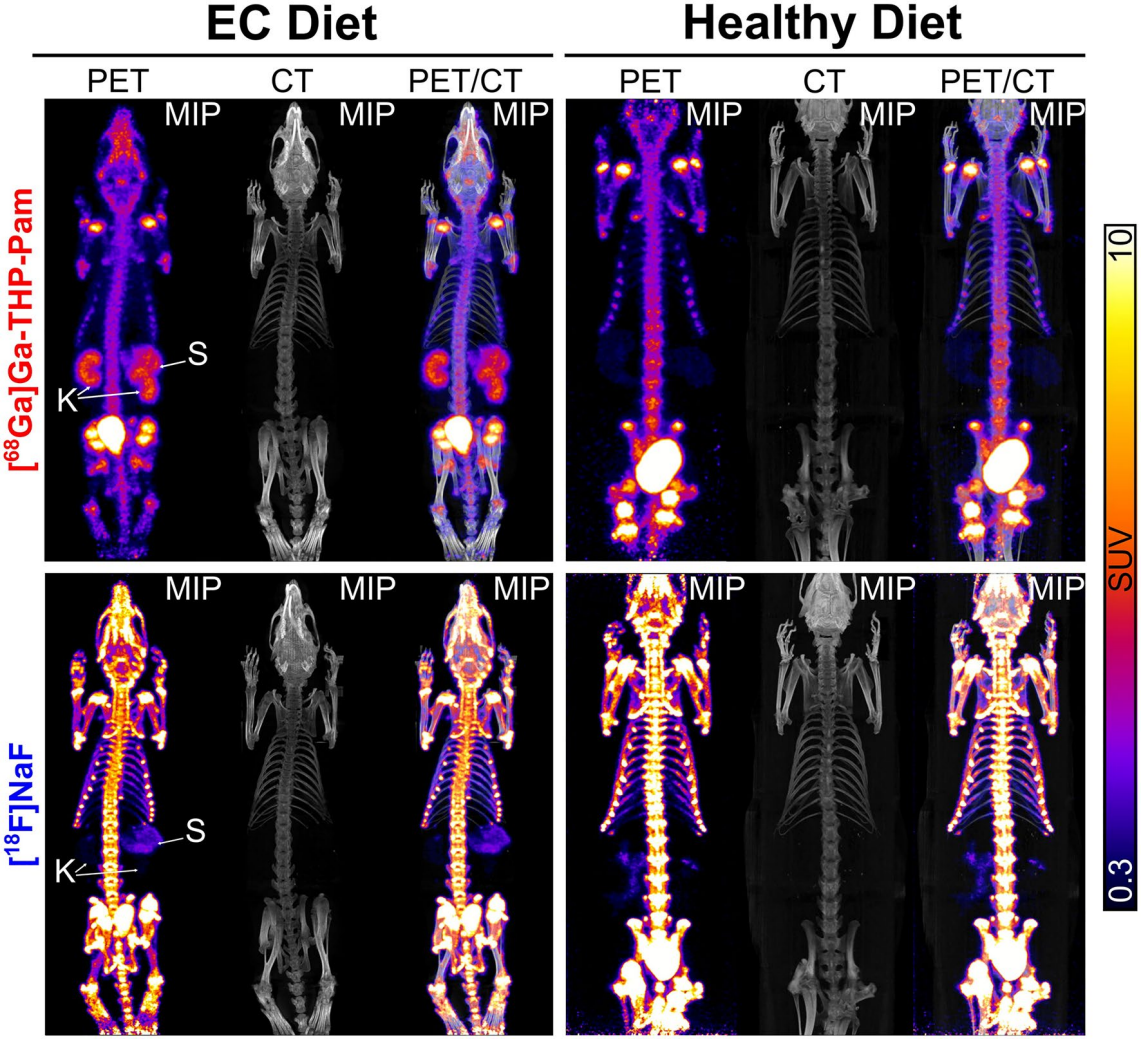
Imaging data of rats with extraosseous calcification and control healthy rats. Maximum intensity projection (MIP) PET, CT and PET/CT images of rats with extraosseous calcification and control rats with both [^68^Ga]Ga-THP-Pam and [^18^F]NaF 60–120 min post-injection. The images of calcified rats are both of the same animal, and the control images are also data from the same animal. *K* kidneys, *S* stomach.

**Figure 3:**
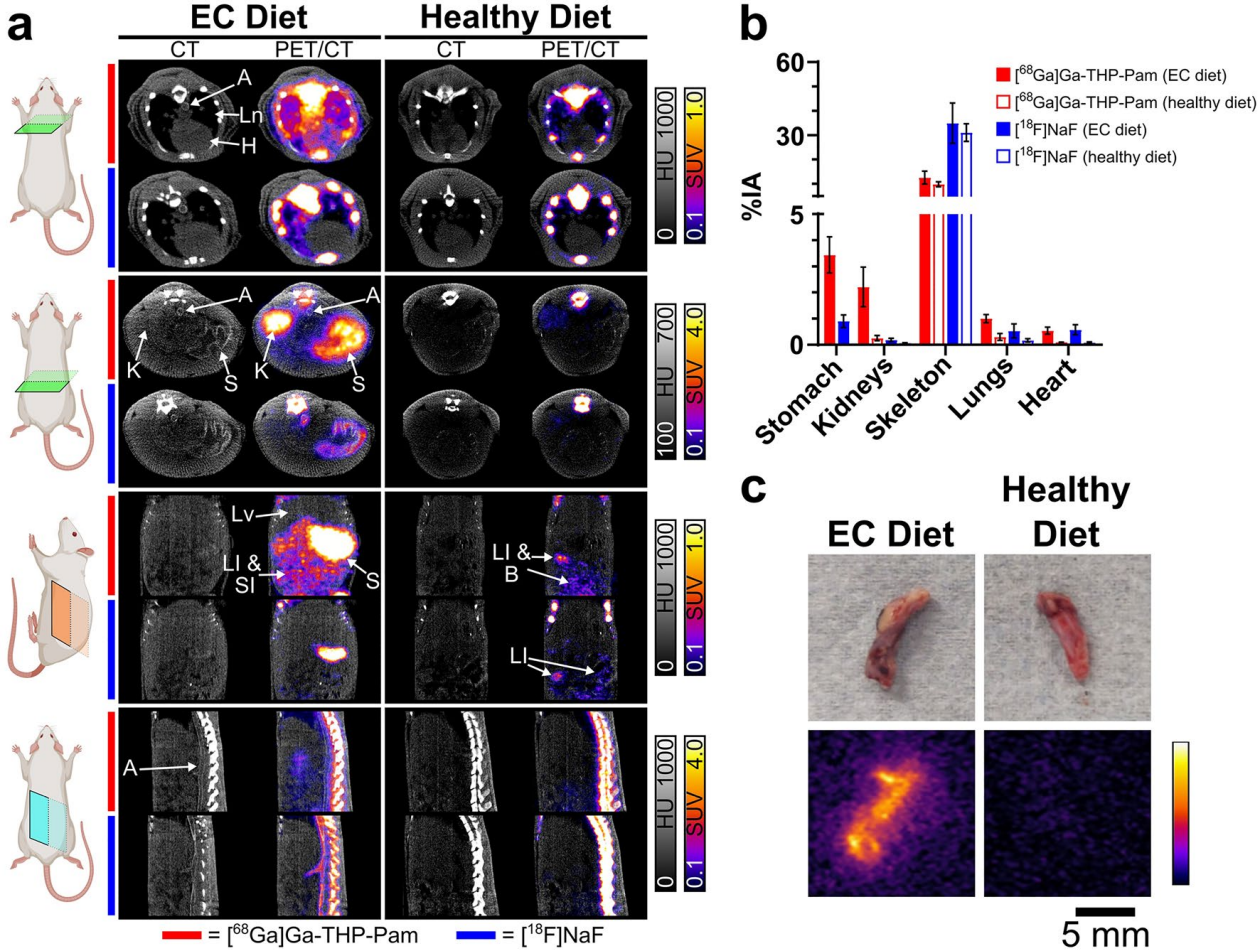
Analysis of PET imaging. (**a**) CT and PET/CT images with [^68^Ga]Ga-THP-Pam (in rows indicated by red bars) and [^18^F]NaF (in rows indicated by blue bars) 60–120 min post-injection. The panels show (from top to bottom): axial view of the heart and lungs; axial view of the stomach and kidneys; coronal view of the abdomen; sagittal view of the abdominal spine and aorta. CT and PET scales are matched for each view. (**b**) Quantified PET data of the %IA in each organ of interest ([^68^Ga]Ga-THP-Pam: n = 4; [^18^F]NaF: n = 3). The entire organ has been included in the ROI, and stomach data from rats fed a healthy diet has been excluded from the data as accurate ROIs could not be drawn due to the stomach not being visible in the images. Data are tabulated in Table S1 (Supplementary Information). (**c**) Ex vivo light images (top) and pseudo-coloured autoradiography (bottom) of abdominal sections from rats injected equal volumes of the same batch of [^68^Ga]Ga-THP-Pam and culled 2 h post-injection, showing higher uptake in the aorta of rats fed the EC diet than in rats fed the healthy diet. *A* aorta, *B* bladder, *H* heart, *K* kidneys, *LI* large intestine, *Ln* lungs, *Lv* liver, *S* stomach, *SI* small intestine.

**Figure 4:**
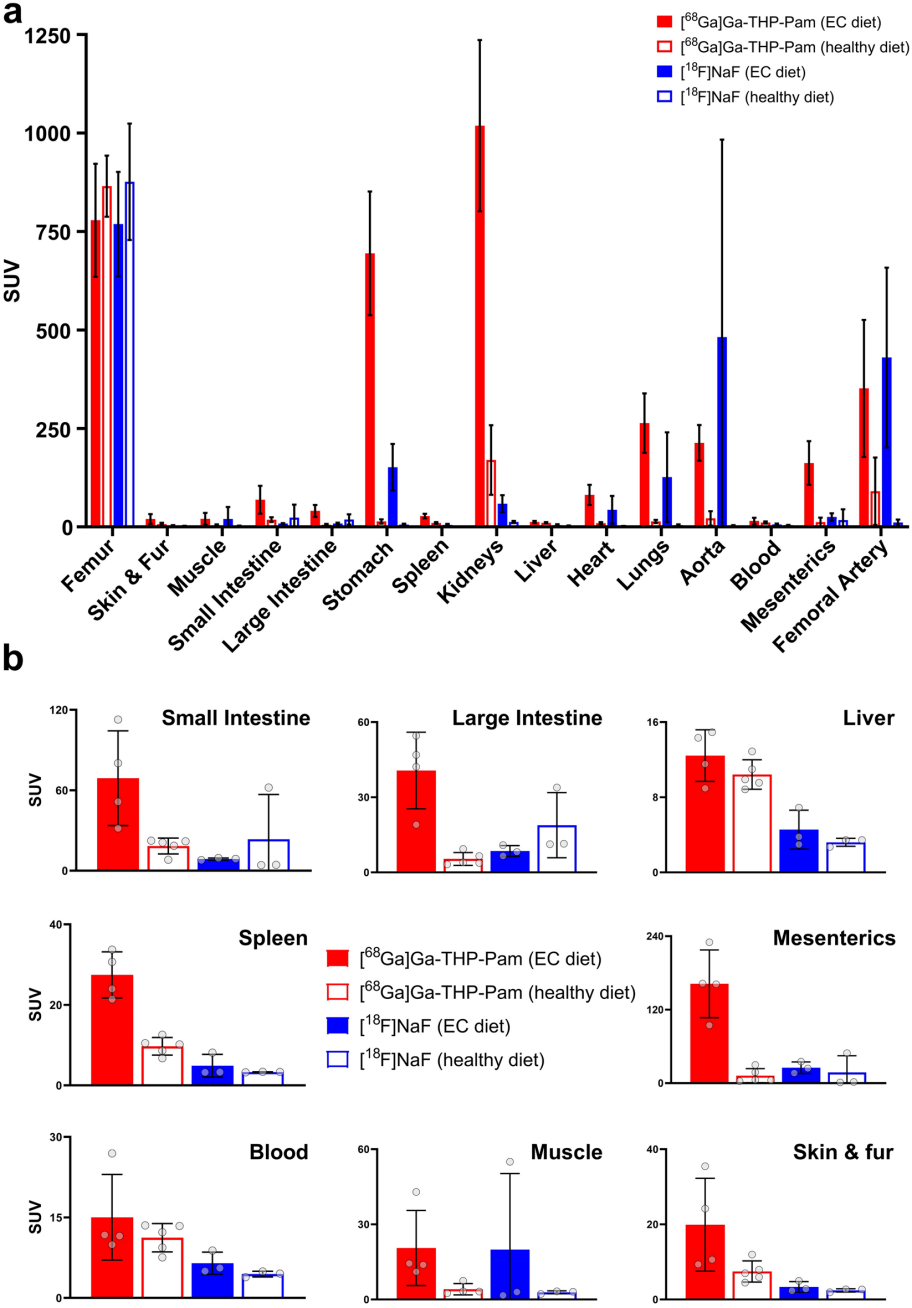
(**a**) Ex vivo biodistribution 2 h post-injection of [^68^Ga]Ga-THP-Pam (EC diet: n = 4; healthy diet: n = 5) and [^18^F]NaF (both diets: n = 3) in rats fed a diet to induce EC and rats fed a healthy diet. Data are tabulated in Table S2 (Supplementary Information). (**b**) Re-scaled ex vivo biodistribution in individual organs from panel a, presented for clarity.

**Figure 5:**
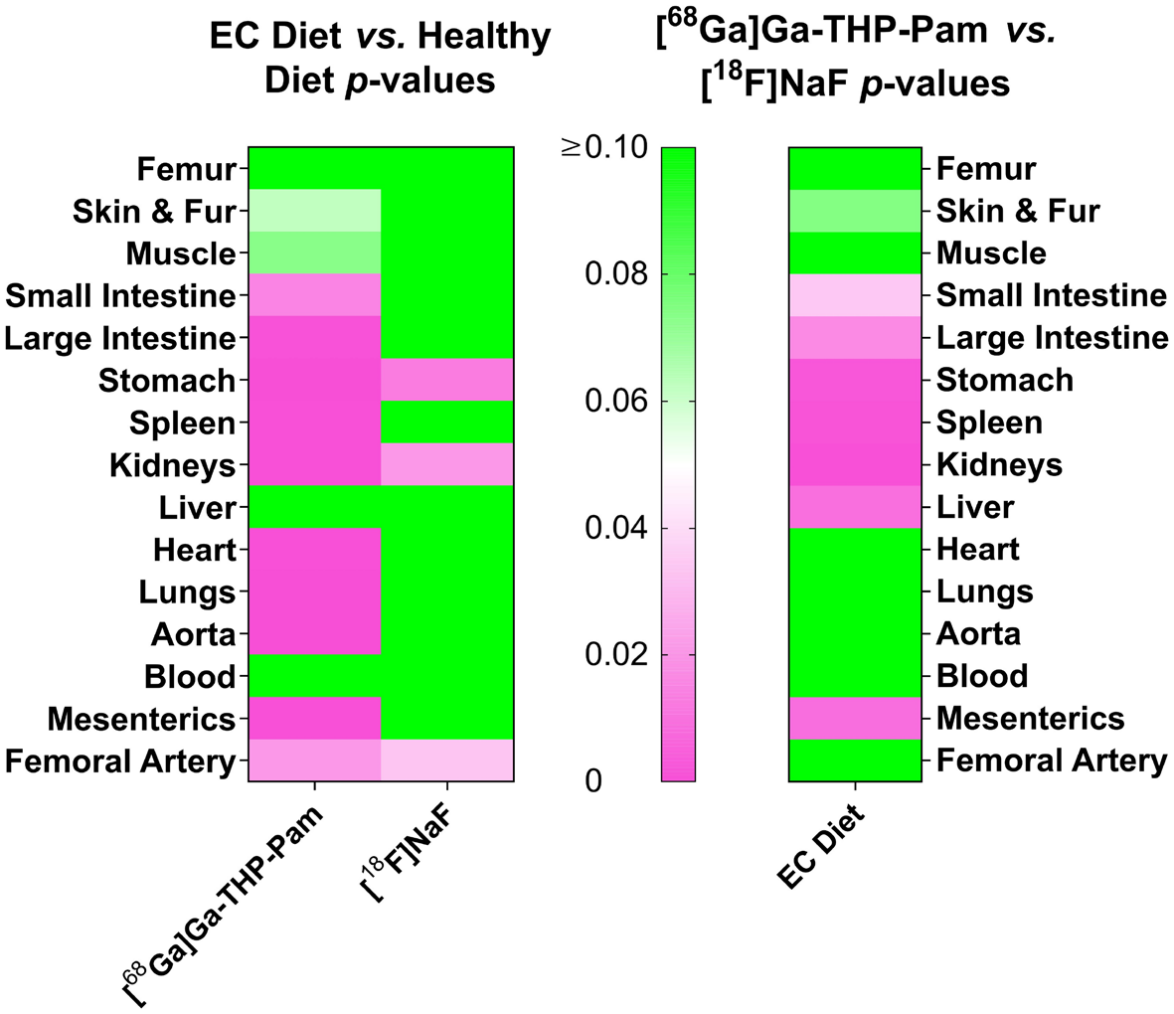
Heat map of *p*-values resulting from unpaired t-tests when comparing the ex vivo biodistribution data of the EC group with the control group with each tracer (left panel) and comparing the two tracers in the EC diet (right panel). The scale has been set to show *p* = 0.05 as white, *p* > 0.05 as green and *p* < 0.05 as pink.

PET/CT imaging with both radiotracers (Figs. 2, 3a,b, Table S1) show equal uptake in the skeleton (Fig. 3b) of both groups—extraosseous calcification diet (EC) and healthy diet—but differences in other organs. For example, both the stomach and kidneys of the rats fed the EC diet demonstrated a higher uptake of [^68^Ga]Ga-THP-Pam (3.44 ± 0.69%IA—stomach; 2.21 ± 0.76—kidneys) than [^18^F]NaF (0.91 ± 0.24%IA—stomach; 0.19 ± 0.06%IA—kidneys). Uptake of both radiotracers in these organs in the healthy diet group were significantly lower. Significant increases in uptake were also seen between the two groups with both tracers in the heart, and with [^68^Ga]Ga-THP-Pam in the lungs. Aortic and stomach calcification was visible by CT and detected with both radiotracers by PET (Fig. 3a,c). Ex vivo biodistribution data at 2 h post-injection presented as standardised uptake value (SUV) (Fig. 4, Table S2) indicated similar trends, with significant differences in more organs (Fig. 4b) and with higher sensitivity (Fig. 5).

### Detection of microcalcifications

**Figure 6:**
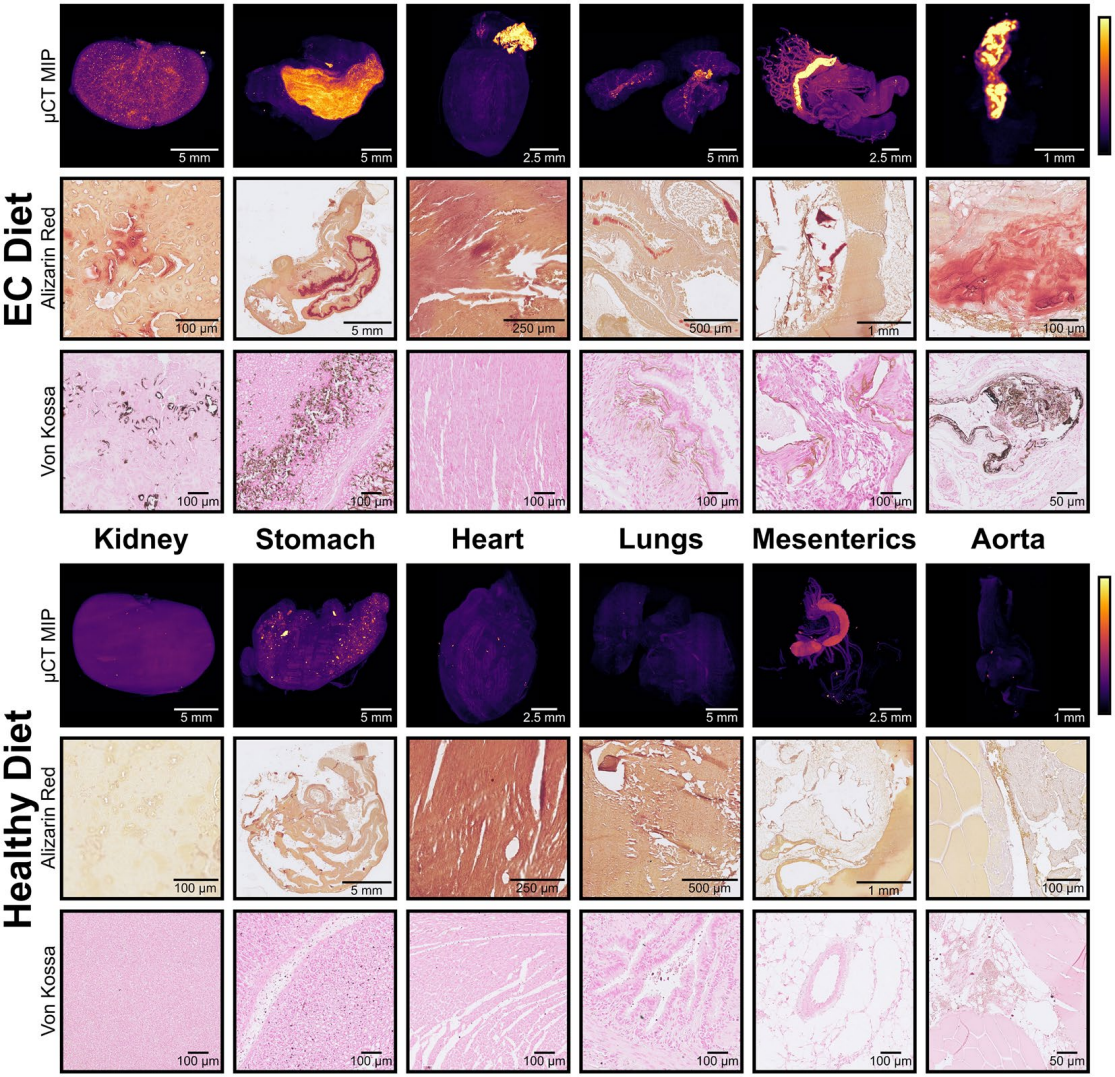
Ex vivo analysis of organs to identify calcification. The top half shows organs from rats fed the EC diet, the bottom half shows organs from rats fed the healthy diet. Each row is labelled with its modality. µCT MIP images show pseudo-coloured whole-organ images, both images for each organ have been calibrated to the same colour scale using the air and paraffin wax as reference points. Alizarin Red and von Kossa images show representative 5 µm slices from the organ stained with Alizarin Red S and von Kossa stains respectively to detect calcium. Both images for each organ are shown at the same scale.

µCT-based 3D X-ray histology (XRH) and conventional histology were used to identify extraosseous calcification in selected organs ex vivo. Animated MIPs and renderings of the XRH data are available online (Supplementary information Table S3). The results (Fig. 6) confirmed the presence of macro- and micro-calcium deposits in the stomach, aorta, kidneys, mesenterics, heart, lungs and aorta.

### Mineral analysis of calcified deposits

**Figure 7:**
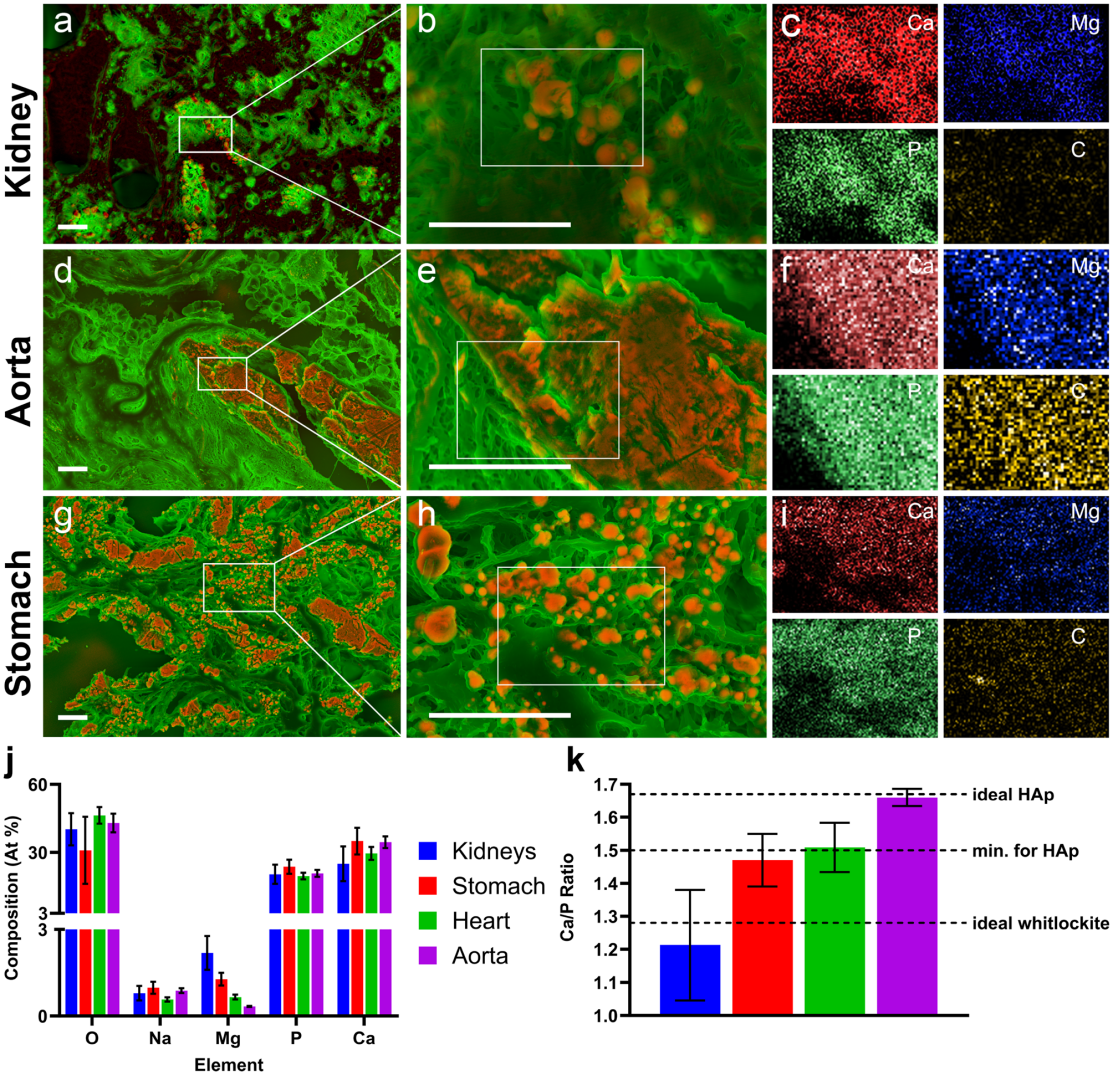
Density-dependent colour SEM (DDC-SEM)^42^ EDX images (panels **a**,**d**,**g**: red/orange dense material (mineral) and green less dense material (organic compounds)) and zoom (panels **b**,**e**,**h**) with calcium-rich mineral shown in red/orange and elemental maps (multi-panels **c**,**f**,**i**) for calcium (red), magnesium (blue), phosphorus (green) and carbon (yellow). (**a**–**c**) Kidneys. (**d**–**f**) Aorta. (**g**–**i**) Stomach. (**j**) EDX results of atomic composition of calcifications found in the kidneys, heart, stomach and aorta. (**k**) Molar calcium-to-phosphorus ratios of mineral present in each organ as determined by SEM/EDX. Data are not absolutely quantitative and should be interpreted only as an indication. The ideal stoichiometric Ca/P ratio of HAp, the minimum ratio required to begin the formation of HAp^43^ and the ideal stoichiometric Ca/P ratio^44^ are indicated in panel k.

Scanning Electron Microscopy (SEM) images from selected key organs (kidney, aorta and stomach) indicated a variety of morphologies which varied by organ (Fig. 7). In the kidneys (Fig. 7a–c), solid calcium mineral was observed to consist of many small calcifications (Fig. 7a), in agreement with the XRH data, and a porous appearance (Fig. 7b). By contrast, the SEM images of the aorta (Fig. 7d–f) show a singular solid plaque with a slab-like appearance. Finally, the stomach (Fig. 7g–i) shows a mixture of these sorts of morphologies.

These data were further analysed for the atomic composition of the calcifications (Fig. 7j–k) using Energy-dispersed X-ray spectroscopy (EDX)—which is semi-quantitative so these data are interpreted only as approximations and not as definitive. Nonetheless, interesting differences between the organs were observed. Firstly, the Ca/P ratios of the stomach (1.47 ± 0.06) and kidneys (1.21 ± 0.16) were not consistent with the theoretical Ca/P ratio of HAp (1.67) and, in the case of the kidneys, the Ca/P ratio was significantly below the reported minimum Ca/P ratio at which HAp is present in biological mineral (1.50)^43^. The aorta however, showed a Ca/P ratio (1.66 ± 0.02) matching the theoretical Ca/P of HAp and in agreement with the highly crystalline structure observed in Fig. 7d,e. Secondly, the levels of magnesium present in the mineral were significantly higher in both the kidneys and stomach than in the heart and aorta.

## Discussion

PET/CT images with [^68^Ga]Ga-THP-Pam of the rats fed the EC diet (Figs. 2, 3a) showed bone and kidney uptake and fast renal/urinary excretion as seen previously in healthy mice^17^, but additionally showed significant uptake in the stomach. A similar pattern of uptake has been reported in a previous study of using BP-based tracers to image calcification^19^. The CT images showed areas of high-density tissue colocalised with the PET signal in the stomach (Fig. 3a), hypothesised to be highly calcified tissue. [^18^F]NaF images in the EC group showed the expected bone and bladder uptake, but no significant amounts of [^18^F]NaF were retained in the kidneys compared to [^68^Ga]Ga-THP-Pam. Additionally, [^18^F]NaF showed some uptake in the stomach in the rats fed the EC diet, but it was significantly lower than with [^68^Ga]Ga-THP-Pam. On the other hand, [^18^F]NaF images of the EC group showed clear uptake in the aorta (Fig. 3a), particularly in the abdominal aorta, which was not visible with [^68^Ga]Ga-THP-Pam. Examination of the CT images (Fig. 3a) showed clear aortic calcification in the EC diet group. The lack of visible [^68^Ga]Ga-THP-Pam uptake is due to limitations in the spatial resolution of ^68^Ga imaging, and uptake of this radiotracer was confirmed via autoradiography (Fig. 3c).

The images of the control group fed a healthy diet demonstrated key differences when compared to those fed the EC diet. First is the lack of signal in the stomach, both by PET and CT, with both tracers. Second is the difference in kidney uptake with [^68^Ga]Ga-THP-Pam, with high signal observed in the EC group *vs*. the healthy control. CT imaging did not show kidney calcification. Heart/lung uptake of [^68^Ga]Ga-THP-Pam and [^18^F]NaF were also increased in the EC group compared to the control, but calcification could not be detected by CT. Less prominent increases in uptake of both tracers were observed across several major organs (Fig. 3a). PET quantification (Fig. 3b) indicated an increased accumulation of both [^68^Ga]Ga-THP-Pam and [^18^F]NaF in the kidneys, lungs and heart of the EC group *vs*. the healthy control.

The ex vivo biodistribution data largely agree with the trends observed by PET, and demonstrated significant differences in other organs. Both tracers had high uptake in the kidneys and stomach of the EC groups compared to the healthy groups, with [^68^Ga]Ga-THP-Pam showing greater uptake than [^18^F]NaF. Similar trends were observed to a lesser extent in several other major organs, with [^68^Ga]Ga-THP-Pam uptake in organs of rats fed the EC diet being significantly higher than in those of rats fed the healthy diet. The heat map of *p*-values from unpaired t-tests (Fig. 5) shows the significant increase in [^68^Ga]Ga-THP-Pam uptake in the majority of internal organs.

While calcification in the rats fed the EC diet was evident from the CT in both the aorta and stomach, it was not visible in other organs and hence the cause of the increased uptake of each tracer could not be determined using this technique. To study these organs, we used XRH allowing detection of micro-calcifications smaller than those able to be detected by preclinical CT^45^. Standard histology of the same samples further confirmed the presence of calcium deposits.

The elemental composition and morphology of these deposits were investigated at higher spatial resolutions using SEM and EDX spectroscopy. Comparisons in the ex vivo biodistribution were made between the aorta—where the differences between [^68^Ga]Ga-THP-Pam and [^18^F]NaF uptake were insignificant—and the stomach and kidneys, where differences in uptake were significant. The slab-like morphology in of the deposits in the aorta is typical of advanced disease and cardiac calcification, likely to be HAp, which forms as the plaque stabilises and becomes less prone to rupture^33^. Both radiotracers showed good binding to these deposits. On the other hand, the porous small deposits observed in the kidneys are typical of earlier stage calcification with a less crystalline structure and can likely be considered amorphous calcium phosphate or whitlockite^34^. The stomach shows a mixture of large slab-like plaques (Fig. 7g) and smaller nodules (Fig. 7h). These smaller irregularly shaped calcifications are typical of whitlockite and unlikely to change as disease advances. For these two organs, [^68^Ga]Ga-THP-Pam showed a significant increased binding vs. [^18^F]NaF.

Elemental analysis of the calcium minerals found in tissues from key organs provided insight into their compositions. As expected, the elemental mapping in Fig. 7c,f,i shows high levels of calcium corresponding to visible areas of calcification in panels b, e and h (from kidney, aorta, and stomach tissue; respectively). However, interestingly, kidney tissue shows increased levels of magnesium relative to carbon (Fig. 7c,j). Magnesium is present in whitlockite (Ca_18_Mg_2_(HPO_4_)_2_(PO_4_)_12_; Ca/P ratio 1.28), which has been identified as the second most abundant mineral in bone^44^, although this has been disputed^46^. Indeed, the dispute claimed that whitlockite is present almost exclusively in pathological calcification and teeth, with high levels detected in calcification of several major arteries. The presence of Mg^2+^ cations has also been reported to inhibit the formation of HAp, stabilising amorphous calcium phosphate and whitlockite^47^, which has been identified in the arteries of CKD patients^31^. Furthermore, these elemental analysis measurements revealed that the Ca/P ratios of the mineral deposits found in kidney, aorta, and stomach tissues deviated from that of HAp (theoretical HAp Ca/P ratio = 1.67), contrasting to that measured for the aorta (1.66 ± 0.02) that matches HAp. Overall, these results strongly suggest that the composition of the calcium minerals varies between these organs, with the aorta mineral matching closely to that of HAp, and other areas of calcification—notably in the kidneys—being consistent with other calcium phosphates with higher Mg^2+^ content and lower Ca/P ratios.

The observed uptake with each tracer in the organs with detected calcification, in combination with the SEM and EDX data and our previous in vitro data, are consistent with the hypothesis that the composition of calcification is diverse, and that [^18^F]NaF—which almost exclusively targets HAp—may not be as well-suited to the imaging of extraosseous calcification as it is to bone. Furthermore, taking into account the reported trend for HAp to form at later stages of calcification, while amorphous calcium phosphate and whitlockite are more prevalent at earlier stages, BP-based imaging agents may not only offer increased sensitivity at later timepoints but potentially even greater increased sensitivity at earlier timepoints of the EC process compared to both CT and [^18^F]NaF PET.

One of the main limitations of the model used is that while it represents severe, more advanced calcification in some organs and milder calcification in others, it does not allow for longitudinal studies in individual animals. Excluding the subcutaneous vitamin D_3_ injections would decelerate the calcification process. Further studies to investigate our hypothesis should focus on the stage of calcification as well as the characterisation of calcification at different stages in various diseases.

Literature covering the analysis of calcifications in different conditions is scarce, however evidence exists to suggest imaging with BP-based agents such as [^68^Ga]Ga-THP-Pam may represent an improvement on the current standards of care. Schlieper et al. demonstrated that in a cohort of uraemia patients with iliac artery calcification, the majority contained either a mixture of HAp and whitlockite or solely whitlockite, whilst no coronary arteries or brachial arteries contained solely HAp^31^. Furthermore, evidence exists of the presence of microcalcification in transthyretin (ATTR) cardiac amyloidosis, which is often imaged with certain [^99m^Tc]Tc-BPs^48^, but is less well detected by [^18^F]NaF^49,50^. Our results suggest further characterisation of these calcifications may aid in understanding the uptake mechanism of different tracers and thus the variability in sensitivity seen in tracers, which could provide the basis for designing trials with new imaging agents.

## Conclusions

We have compared the PET imaging performance of a BP ([^68^Ga]Ga-THP-Pam) to the clinical PET tracer ([^18^F]NaF) in a model of EC in rats. The results showed a significantly increased uptake of both tracers in several major organs in the EC group rats, with the increase in uptake being greater with [^68^Ga]Ga-THP-Pam than with [^18^F]NaF. X-ray and histology studies confirmed the presence of calcification in these organs. Finally, the composition and morphology of the calcification were studied by SEM and EDX, which demonstrated that in organs where both radiotracers performed similarly by PET, the composition more closely matched theoretical HAp. Yet, in organs in which [^68^Ga]Ga-THP-Pam detected calcification more sensitively than [^18^F]NaF, the composition varied away from theoretical HAp. Therefore, we propose that BP-based tracers may be more appropriate for imaging EC than the HAp-specific [^18^F]NaF, offering the possibility of earlier detection than [^18^F]NaF and CT, before mineral matures into HAp and reaches sufficient density for CT detection.

## Materials and methods

### Materials

All chemicals were purchased from commercial sources unless stated otherwise. Gallium-68 was eluted as [^68^Ga]GaCl_3_ from an Eckert & Ziegler (Germany) ^68^Ge/^68^Ga generator in ultra-pure HCl (5 mL, 0.1 M) manufactured to good manufacturing practice (GMP) requirements (ABX, Germany). [^18^F]NaF in H_2_O was purchased from Alliance Medical, UK.

### Synthesis of THP-Pam

THP-Pam was synthesised using an adapted version of our previously published method^17^. In brief, THP-NCS (10.0 mg, 10.4 µmol, CheMatech, France) was dissolved in water (400 µL) and triethylamine (42.4 µL, 30.8 mg, 0.304 mmol, Merck Life Science UK Limited, UK). Pamidronate disodium (29.0 mg, 104 µmol, synthesised as previously published^17^) was dissolved in water (400 µL) and triethylamine (42.4 µL, 30.8 mg, 0.304 mmol, Merck Life Science UK Limited, UK). The THP-NCS solution was added to the pamidronate solution and stirred at 90 °C for 3 h. The crude solution was purified by semi-preparative HPLC: column: Agilent ZORBAX Eclipse XDB-C18 (9.4 × 250 mm, 5 μm); solvent A: water + 0.1% formic acid; solvent B: acetonitrile + 0.1% formic acid; flow rate = 4 mL min^–1^; 0–5 min = 5% B, 5–40 min = 5–20% B, 40–41 min = 20–95% B, 41–45 min = 95% B, 45–46 min = 95–5% B, 46–50 min = 5% B. The identity of the product was confirmed by LC/MS, ^1^H NMR and ^31^P NMR.

### Radiochemistry

[^68^Ga]Ga-THP-Pam was synthesised using our previously published method^17^, giving a product with a concentration of 82.4–176.0 MBq mL^–1^ at end of synthesis, and filtered through a 0.22 µm syringe filter prior to injection into animals.

[^18^F]NaF was diluted to a concentration of 115.1–136.3 MBq mL^–1^ at the time of dilution using 0.9% sterile saline and filtered through a 0.22 µm syringe filter prior to injection into animals.

### Rat model of extraosseous calcification (EC) and healthy diet control

A summary of the following set-up of the calcification model and PET imaging/ex vivo biodistribution protocol is shown in Fig. S1:

- Extraosseous calcification (EC) group: Sprague Dawley rats (n = 8, male, aged 21–27 days on arrival) were acclimatised for 7 days. The rats were then fed a specialised diet (manufactured by LBS Biotechnology, UK, supplied by Special Diet Services, UK) for 12 days (days 0–11 inclusive). The diet consists of a vitamin K-deficient normal rodent diet supplemented with warfarin (3 mg g^–1^ food, TCI UK Ltd, UK) and vitamin K_1_ (1.5 mg g^–1^ food, Apollo Scientific, UK). For the final 4 days of the diet (days 8–11 inclusive), each rat was injected subcutaneously with cholecalciferol (vitamin D_3_, 5 mg kg^–1^ day^–1^). The stock solution of cholecalciferol was prepared by addition of cholecalciferol (33.0 mg, 85.8 μmol, Sigma-Aldrich, UK) to absolute ethanol (200 μL) and Kolliphor EL (1.4 mL, Sigma-Aldrich, UK), which was mixed in the dark for 15 min. d-(+)-glucose (750 mg, 4.16 mmol, Sigma-Aldrich, UK) was dissolved in water (18.4 mL) and added to the cholecalciferol solution and mixed in the dark for 15 min. The stock solution was stored in the dark at 4 °C for up to 3 days. Rats were returned to a healthy diet at the end of day 11.
- Healthy diet (control) group: Control rats were Sprague–Dawley rats (n = 8, male, aged 21–48 days upon arrival) which were fed a healthy diet and not injected with cholecalciferol. Cages of animals were selected to be in the test group or control group at random prior to arrival. Researchers were not blinded to whether the animals were fed a normal diet or not.

### PET/CT imaging and biodistribution studies

On day 11, rats were anaesthetised by inhalation of isoflurane (1.5–4% in oxygen) and the tail vein was cannulated using sterile saline. Each rat was injected intravenously with [^68^Ga]Ga-THP-Pam (100 ± 20 μL, 1.0–6.6 MBq). The rat was maintained under anaesthetic on a warm bed to maintain body temperature for 30 min. The rat was placed in a Mediso nanoScan® PET/CT scanner, where anaesthesia was maintained, and the bed was heated to maintain normal body temperature and CT (55 kVp) was performed. At 1 h post-injection, 1 h PET acquisition (3 × 20 min fields of view, 1:5 coincidence mode; 5-ns coincidence time window) was performed. On day 12, the rats were anaesthetised by inhalation of isoflurane (1.5–4% in oxygen) and the tail vein was cannulated. Each rat was injected intravenously with [^18^F]NaF in sterile saline (100 ± 15 μL, 2.7–11.7 MBq) and imaged at 1 h post-injection on the PET/CT scanner using the same procedure used on day 11. At the end of the scan, the animal was culled at 2 h post-injection for ex vivo biodistribution studies. Additionally, non-imaging rats were anaesthetised by inhalation of isoflurane (1.5–4% in oxygen) and the tail vein was cannulated. The rats were injected intravenously with [^68^Ga]Ga-THP-Pam (100 ± 5 μL, 3.9–10.9 MBq). The rats were maintained under anaesthetic on a warm bed to maintain body temperature for 2 h and culled at 2 h post-injection for ex vivo biodistribution studies. Organs were harvested, weighed, and counted with a gamma counter along with standards prepared from injected material. Organs and vasculature of interest (heart, lungs, stomach, kidneys, aorta, mesenterics and femoral artery) were fixed in 10% neutral-buffered formalin, after weighing, and subsequently embedded in paraffin for further analysis. For autoradiography, sections of abdominal aorta (approximately 1 cm long, centred on the branching points of the celiac and superior mesenteric arteries) from four rats (two fed the EC diet, two fed a healthy diet) were collected 2 h post-injection with the same volume (100 ± 10 μL) of the same stock of [^68^Ga]Ga-THP-Pam during biodistribution studies. The aortas were placed under a PerkinElmer MultiSensitive Phosphor Screen (12.5 × 25.2 cm) for 5 min and the film was transferred to a Typhoon 8600 Variable Mode Imager. The results were processed using the open-source image processing and analysis package Fiji^51^. The image was pseudo-coloured using the mpl-inferno scale.

### μCT-based 3D X-ray histology (XRH)

µCT was performed at the 3D X-ray Histology facility, µ-VIS X-ray Imaging Centre at the University of Southampton (www.xrayhistology.org). 3D X-ray histology (XRH) is a µCT-based imaging technique that allows non-destructive 3D (volume) visualisation of standard formalin-fixed, paraffin-embedded (FFPE) biopsy specimens and can be seamlessly integrated into conventional histology workflows, enabling non-destructive three-dimensional (3D) X-ray histology. FFPE tissue samples were scanned using a custom designed µCT scanner optimised for XRH (based on the Nikon XTH225ST system, Nikon Metrology UK Ltd.). Imaging was conducted at 80 kVp. Some of the imaging parameters such a as the number of projections, voxel size, and the frames per projection were optimised for each organ based on several factors including sample size and position of the tissue on the cassette. Table S4 lists all critical parameters for each organ. Scanning was conducted on the histology cassette (blocks were not dewaxed), without the addition of X-ray contrast agents to allow for further analysis by means of conventional histology and consequent correlative imaging.

Upon acquisition, the projection data were reconstructed using conventional filtered back projection into 32-bit floating-point volumes using Nikon’s CT reconstruction. Following CT reconstruction, the data volumes were converted to 16-bit and resliced (re-oriented in silico) so that a scroll through the stack along the Z-direction emulates the physical histology slicing of the tissue. This way, any XY single slice through the image stack is parallel to the histology cassette and the XY slice-scroll runs from the wax-block’s surface towards the cassette (similarly to the knife on physical sectioning; see Table S3) and manually cropped to the boundaries of the tissue within the scan using Fiji^15^. A 3D rendered video was also produced and can be viewed at https://figshare.com/s/43d83b118d78bf22da97.

### Histological staining

Histological analysis was performed by IQPath, Department of Neurodegenerative Disease, UCL. Von Kossa staining counterstained with nuclear fast red and Alizarin Red S staining were performed on 5 µm slices of the tissues. Slices were scanned on a Hammatsu S360 digital slide scanner at × 40 magnification and visualised using NZConnect software.

### SEM/EDX

For scanning electron microscopy analysis, paraffin wax was removed using pure xylene for two 10-min intervals. The slides were then mounted on sample holders using double sided carbon adhesive tape, painted with silver conductive paint, and coated with a 5 nm carbon layer. A Hitachi S-3499N at Wolfson Lab in the Archaeology Department of University College London (UCL) and a Zeiss Leo 1525 Gemini at Electron microscope suite in the Materials Science department of Imperial College London were used for imaging. The Energy Dispersive X-ray spectroscopy (EDX) analysis was applied using an Oxford Instrument EDX detector integrated into the microscope. A 10 kV accelerating voltage and 10 mm working distance settings are used to obtain high-resolution images; the secondary electron (SE) mode was used to get topographic information of the samples, while the backscatter electron detector (BSE) mode enables the differentiation of organic and inorganic materials. The Zeiss obtains two SE images at different depths, which helps us to build a better density-dependent colour SEM (DDC-SEM)^52^ image of the minerals and the tissue. EDX is performed on selected areas or points to get the elemental composition and distribution.

### Statistics

Statistical analyses were performed using GraphPad Prism 9 (GraphPad Software Inc., USA) software. Data are presented as mean ± 1 standard deviation. Comparisons between results were analysed using unpaired t-tests, with a *p*-value < 0.05 considered statistically significant. Outlying data points in ex vivo biodistribution studies may derive from suspected cross contamination of samples during experiments and were excluded using Tukey’s Fences test.

### Ethics approval

Animal imaging studies were ethically reviewed and carried out in accordance with the Animals (Scientific Procedures) Act 1986 (ASPA) UK Home Office regulations governing animal experimentation. Animal studies were carried out in accordance with the United Kingdom’s Research Councils and Medical Research Charities’ guidelines on ‘Responsibility in the Use of Animals in Bioscience Research’. These studies were performed under the legal authority conferred by the United Kingdom’s Home Office Project Licence PP8261525. All Project licences are reviewed and approved by King’s College London’s Animal Welfare and Ethical Review Body prior to final submission to the United Kingdom’s Home Office. The study is reported in accordance with ARRIVE guidelines.

### Supplementary Information

Supplementary Information.

## Data Availability

The datasets generated during and/or analysed during the current study are available from the corresponding author on reasonable request.
