# Supplementary material for: 68Ga-bisphosphonates for the imaging of extraosseous calcification by positron emission tomography
Source: Sci Rep. 2023 Sep 5;13:14611. doi: 10.1038/s41598-023-41149-7 (PMC10480432; doi:10.1038/s41598-023-41149-7)
Supplement: Supplementary file 1 — Supplementary Information. [file 41598_2023_41149_MOESM1_ESM.docx]

**Supplementary Information for:**

**^68^Ga-Bisphosphonates for the Imaging of Extraosseous Calcification by Positron Emission Tomography**

George P. Keeling^a^, Friedrich Baark^a^, Orestis L. Katsamenis^b^, Jing Xue^c^, Philip J. Blower^a^, Sergio Bertazzo^c^, Rafael T. M. de Rosales^a*^

1. Department of Imaging Chemistry & Biology, School of Biomedical Engineering & Imaging Sciences, King’s College London, St Thomas’ Hospital, London, UK, SE1 7EH
2. µ-VIS X-Ray Imaging Centre, Faculty of Engineering and Physical Sciences, Highfield Campus, University of Southampton, Southampton, UK, SO17 1BJ
3. Department of Medical Physics & Biomedical Engineering, University College London, Malet Place Engineering Building, London, UK, WC1E 6BT

**Figure S1**. Schedule of PET imaging (top) and *ex vivo* biodistribution (bottom) of rats fed a healthy diet (n = 8) or a EC diet (n = 8). Overview of the schedule of set-up, imaging and biodistribution of rats in the EC study. Red arrows = rats injected with [^68^Ga]Ga-THP-Pam. Blue arrows = rats injected with [^18^F]NaF.

| %IA | [^68^Ga]Ga-THP-Pam | | | | [^18^F]NaF | | |
| --- | --- | --- | --- | --- | --- | --- | --- |
|  | EC Diet (n = 4) | | | Healthy Diet (n = 4) | EC Diet (n = 3) | | Healthy Diet (n = 3) |
|  | mean ± SD | p-value | | mean ± SD | mean ± SD | p-value | mean ± SD |
|  |  | ^A^ | ^B^ |  |  | ^c^ |  |
| Stomach | 3.44 ± 0.69 | N/A | ** | N/A | 0.91 ± 0.24 | N/A | N/A |
| Left Kidney | 2.15 ± 0.57 | *** | ** | 0.29 ± 0.20 | 0.19 ± 0.07 | * | 0.07 ± 0.02 |
| Right Kidney | 2.28 ± 0.95 | ** | * | 0.22 ± 0.01 | 0.19 ± 0.05 | * | 0.07 ± 0.00 |
| Skeleton | 12.57 ± 2.62 | ns | ** | 9.85 ± 1.04 | 34.95 ± 8.28 | ns | 31.06 ± 3.63 |
| Lungs | 1.00 ± 0.15 | *** | * | 0.30 ± 0.13 | 0.53 ± 0.27 | ns | 0.17 ± 0.06 |
| Heart | 0.54 ± 0.13 | *** | ns | 0.10 ± 0.00 | 0.58 ± 0.19 | * | 0.09 ± 0.03 |

**Table S1**. Percentage of the injected activity to accumulate in each ROI 60–120 min post-injection, based on the ROI analysis of the PET-CT images. Stomach data are not included for control animals due to difficulty drawing an accurate ROI because of the lack of CT signal. Significance was calculated using an unpaired t-test. A = *p*-value with respect to [^68^Ga]Ga-THP-Pam Healthy Diet group. B = *p*-value with respect to [^18^F]NaF EC Diet group. C = *p*-value with respect to [^18^F]NaF Healthy Diet Group. ns = not significant; * = *p* ≤ 0.05; ** = *p* ≤ 0.01; *** = *p* ≤ 0.001.

| %IA g^–1^ | [^68^Ga]Ga-THP-Pam | | | | [^18^F]NaF | | |
| --- | --- | --- | --- | --- | --- | --- | --- |
|  | EC Diet (n = 4) | | | Healthy Diet (n = 5) | EC Diet (n = 3) | | Healthy Diet (n = 3) |
|  | mean ± SD | p-value | | mean ± SD | mean ± SD | p-value  ^C^ | mean ± SD |
|  |  | ^A^ | ^B^ |  |  |  |  |
| Femur | 6.00 ± 0.30 | ** | ns | 3.22 ± 1.09 | 5.48 ± 0.74 | ** | 2.48 ± 0.47 |
| Skin & Fur | 0.16 ± 0.11 | * | ns | 0.03 ± 0.01 | 0.02 ± 0.01 | * | 0.01 ± 0.00 |
| Muscle | 0.17 ± 0.13 | ns | ns | 0.03 ± 0.03 | 0.14 ± 0.17 | ns | 0.01 ± 0.00 |
| Small Intestine | 0.55 ± 0.23 | ** | * | 0.07 ± 0.03 | 0.06 ± 0.00 | ns | 0.06 ± 0.07 |
| Large Intestine | 0.34 ± 0.15 | ** | * | 0.02 ± 0.01 | 0.06 ± 0.01 | ns | 0.05 ± 0.03 |
| Stomach | 5.36 ± 0.74 | *** | *** | 0.05 ± 0.02 | 1.08 ± 0.33 | * | 0.02 ± 0.01 |
| Spleen | 0.23 ± 0.08 | ** | * | 0.03 ± 0.01 | 0.03 ± 0.02 | ns | 0.01 ± 0.00 |
| Kidneys | 7.90 ± 1.26 | *** | *** | 0.54 ± 0.19 | 0.42 ± 0.13 | * | 0.03 ± 0.01 |
| Liver | 0.10 ± 0.03 | * | * | 0.04 ± 0.01 | 0.03 ± 0.01 | * | 0.01 ± 0.00 |
| Heart | 0.68 ± 0.30 | ** | ns | 0.03 ± 0.01 | 0.31 ± 0.20 | ns | 0.01 ± 0.00 |
| Lungs | 2.05 ± 0.56 | *** | ns | 0.05 ± 0.01 | 0.89 ± 0.65 | ns | 0.01 ± 0.00 |
| Aorta | 1.69 ± 0.45 | *** | ns | 0.08 ± 0.04 | 3.48 ± 3.01 | ns | 0.01 ± 0.00 |
| Blood | 0.13 ± 0.08 | ns | ns | 0.04 ± 0.01 | 0.05 ± 0.01 | * | 0.01 ± 0.00 |
| Mesenterics | 1.35 ± 0.60 | ** | * | 0.04 ± 0.03 | 0.18 ± 0.05 | ns | 0.05 ± 0.06 |
| Femoral Artery | 2.85 ± 1.59 | * | ns | 0.30 ± 0.19 | 3.08 ± 1.36 | * | 0.03 ± 0.02 |
| Urine | 72.38 ± 57.02 | ns | ns | 99.60 ± 59.76 | 4.15 ± 3.81 | ns | 12.38 ± 3.78 |
| Tail | 1.04 ± 0.22 | ns | ns | 1.01 ± 0.52 | 1.45 ± 0.32 | * | 0.47 ± 0.05 |

**Table S2.** Biodistribution of [^68^Ga]Ga-THP-Pam and [^18^F]NaF in rats fed a diet to induce EC and rats fed a healthy diet. Significance was calculated using an unpaired t-test. A/B/C represent p-values of the radiotracer and diet group of that column, in comparison with: ^A^ = [^68^Ga]Ga-THP-Pam Healthy Diet group. ^B^ = [^18^F]NaF EC Diet group. ^C^ = [^18^F]NaF Healthy Diet Group. ns = not significant; * = p ≤ 0.05; ** = p ≤ 0.01; *** = p ≤ 0.001.

| Group | Organ | Organ ID | Video link |
| --- | --- | --- | --- |
| EC Diet | Aorta | 4P | <https://figshare.com/s/ce48ad47624e58322620> |
| Healthy Diet | Aorta | 8P | <https://figshare.com/s/9a79bde69cc4e2f19c5f> |
| EC Diet | Heart | 13N | <https://figshare.com/s/92d798012874746c05cc> |
| Healthy Diet | Heart | 15N | <https://figshare.com/s/8e2ffea323e39eb79078> |
| EC Diet | Kidney | 4K | <https://figshare.com/s/94d9848a40dcd37203a0> |
| Healthy Diet | Kidney | 8K | <https://figshare.com/s/ce48ad47624e58322620> |
| EC Diet | Lungs | 13O | <https://figshare.com/s/3e37f93eca63fe87cb67> |
| Healthy Diet | Lungs | 15O | <https://figshare.com/s/8e2ffea323e39eb79078> |
| EC Diet | Mesenterics | 4S | <https://figshare.com/s/077f7dc2f06e18f93b0a> |
| Healthy Diet | Mesenterics | 8S | <https://figshare.com/s/ef085fcbe15891af436b> |
| EC Diet | Stomach | 4I | <https://figshare.com/s/bca71661eb1c017cf1da> |
| Healthy Diet | Stomach | 8I | <https://figshare.com/s/35642a0e0e8dd2f3ea8b> |

**Table S3**. Links to videos scrolling through formalin-fixed, paraffin-embedded organs parallel to the histological cassette.

|  | Heart | Stomach | Mesenterics | Lung | Kidney | Aorta |
| --- | --- | --- | --- | --- | --- | --- |
| Energy (kVp) | 80 | 80 | 80 | 80 | 80 | 80 |
| Power (W) | 8.48 | 8.48 | 8.48 | 8.48 | 6 | 6 |
| Voxel edge (mm) | 0.0072 | 0.0120 | 0.0120 | 0.0120 | 0.0125 | 0.0060 |
| Projections | 5001 | 4001 | 4001 | 4001 | 2601 | 2601 |
| Frames per projection | 4 | 4 | 4 | 4 | 8 | 8 |
| Geometric Magnification | 20.871 | 12.49 | 12.49 | 12.49 | 11.998 | 24.981 |
| Estimated scan time (hh:mm) | 02:40 | 02:07 | 02:07 | 02:07 | 03:15 | 03:15 |

**Table S4**. XRH Imaging parameters for each FFPE organ sample.
